# MicroRNAs in Cancer Treatment-Induced Cardiotoxicity

**DOI:** 10.3390/cancers12030704

**Published:** 2020-03-17

**Authors:** Laura Pellegrini, Sara Sileno, Marco D’Agostino, Eleonora Foglio, Maria Cristina Florio, Vincenzo Guzzanti, Matteo Antonio Russo, Federica Limana, Alessandra Magenta

**Affiliations:** 1 Institute of Oncology Research (IOR), 6500 Bellinzona, Switzerland; laura_pellegrini@hotmail.it; 2 Istituto Dermopatico dell’Immacolata, IDI-IRCCS, Experimental Immunology Laboratory, Via dei Monti di Creta 104, 00167 Rome, Italy; sara.sileno@libero.it (S.S.); marcodagostino86@hotmail.it (M.D.); 3 Department of Experimental Medicine, Sapienza University of Rome, 00161 Rome, Italy; eleonora.foglio83@gmail.com; 4 Laboratory of Cardiovascular Science, National Institute on Aging, National Institutes of Health, Baltimore, MD 21224, USA; cristina.florio@nih.gov; 5 Istituto Dermopatico dell’Immacolata, IDI-IRCCS, 00167 Rome, Italy; vguzzanti@yahoo.it; 6 IRCCS San Raffaele Pisana and MEBIC Consortium, 00166 Rome, Italy; matteo.russo@sanraffaele.it; 7 San Raffaele Open University, 00166 Rome, Italy; fe_limana@hotmail.com; 8 Laboratory of Cellular and Molecular Pathology, IRCCS San Raffaele Pisana, 00166 Rome, Italy

**Keywords:** microRNAs, cancer therapy, cardiovascular diseases, cardiotoxicity

## Abstract

Cancer treatment has made significant progress in the cure of different types of tumors. Nevertheless, its clinical use is limited by unwanted cardiotoxicity. Aside from the conventional chemotherapy approaches, even the most newly developed, i.e., molecularly targeted therapy and immunotherapy, exhibit a similar frequency and severity of toxicities that range from subclinical ventricular dysfunction to severe cardiomyopathy and, ultimately, congestive heart failure. Specific mechanisms leading to cardiotoxicity still remain to be elucidated. For instance, oxidative stress and DNA damage are considered key players in mediating cardiotoxicity in different treatments. microRNAs (miRNAs) act as key regulators in cell proliferation, cell death, apoptosis, and cell differentiation. Their dysregulation has been associated with adverse cardiac remodeling and toxicity. This review provides an overview of the cardiotoxicity induced by different oncologic treatments and potential miRNAs involved in this effect that could be used as possible therapeutic targets.

## 1. Introduction

miRNAs are short non-coding RNA molecules of 21–23 nucleotides that modulate the stability and/or the translational efficiency of target messenger RNAs. miRNAs have been shown to regulate most biological processes, including differentiation, proliferation, development, migration, and apoptosis (for extensive reviews on miRNA regulation and biogenesis, see [1,2,3]). Recently their use as biomarkers has strongly developed, since miRNAs are not only intracellular molecules, but also are detectable outside the cells in body fluids (e.g., in serum, plasma, saliva, urine, and milk) [4]. Further, they are protected from RNase degradation, since they are contained in small membranous vesicles (e.g., exosomes, exosome-like vesicles, apoptotic bodies, and microparticles), packaged within HDL-cholesterol, or linked to RNA-binding proteins [4]. Given the robust stability of miRNAs in blood, circulating miRNAs have been used as excellent biomarkers in different studies and can be used as biomarkers for cardiovascular diseases (CVDs) [5]. Moreover, miRNA deregulation is often associated with tumor progression, and many anticancer treatments affect miRNA expression. In this review we aimed to discuss relevant miRNAs modulated by therapies for cancer that have been demonstrated to be involved in CVD. In the following paragraphs, we provide an overview of the most important anticancer treatments that are known to induce cardiotoxicity.

## 2. Anticancer Treatment and Cardiotoxicity

Anticancer treatment has accomplished remarkable progress in the last century resulting in improved quality of life and survival rates of cancer patients. These advances in cancer treatment, however, have been often accompanied by therapy-related complications, including secondary side effects on the whole organism [6].

**Figure 1:**
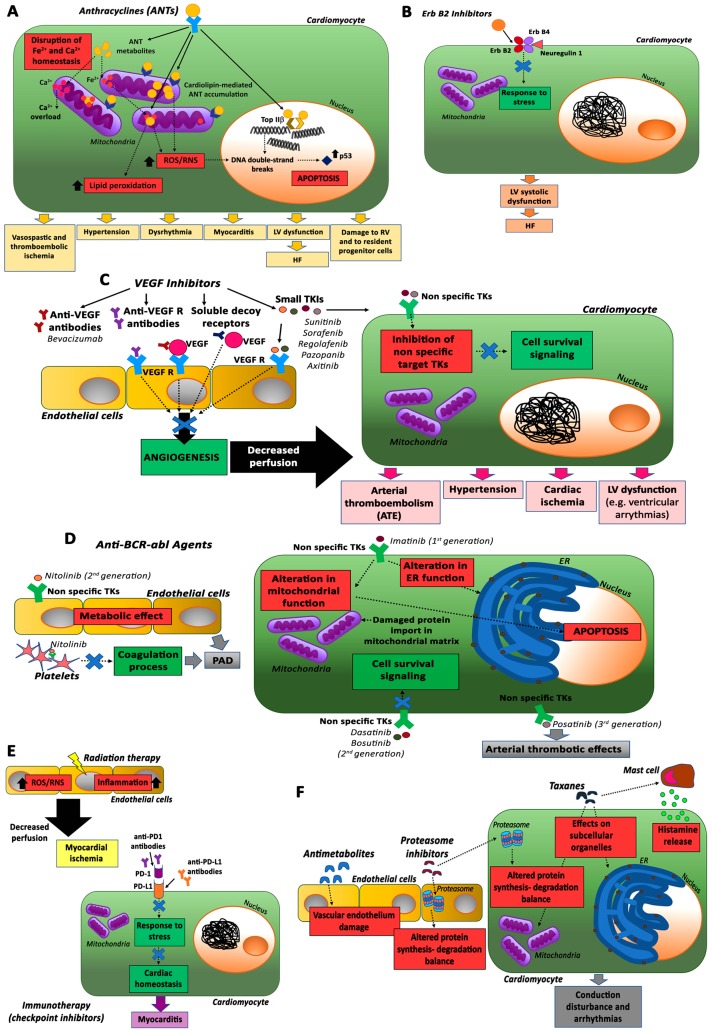
Mechanisms of cardiotoxicity induced by different classes of anticancer therapies. Treatment for cancer diseases can adversely affect both the heart and the vasculature, leading to death of cardiomyocytes, endothelial dysfunction, and, consequently, different cardiovascular complications. Common cellular targets and pathways involved in cardiotoxicity linked to anthracyclines (**A**), ErbB2 inhibitors (**B**), VEGF inhibitors (**C**), anti-BCR-Abl agents (**D**), radiation therapy and immunotherapy (in particular checkpoint inhibitors) (**E**), and other commonly used, less specific antineoplastic drugs (antimetabolites, proteasome inhibitors, and taxanes) (**F**) are schematically illustrated. Abbreviations: ANTs, anthracyclines; TopIIβ, topoisomerase IIβ; ROS, reactive oxygen species; RNS, reactive nitrogen species; LV, left ventricular; RV, right ventricular; HF, heart failure; ErbB2/ErbB4, human epidermal growth factor receptor 2/4; VEGF, vascular endothelial growth factor; R, receptor; TKs, tyrosine kinases; TKIs, tyrosine kinase inhibitors; ER, endoplasmic reticulum; PAD, peripheral artery disease; PD-1, programmed cell death protein 1; PD-L1, programmed cell death protein ligand 1.

The most commonly used cancer therapeutics in modern medicine include the traditional surgery, radiotherapy, and conventional chemotherapy approaches; moreover, two new therapeutic modalities have been introduced in recent decades, namely molecularly targeted therapy and immunotherapy [7], as summarized in Figure 1. Traditional chemotherapy agents consist of non-specific cytotoxic treatments (e.g., alkylating agents and antimetabolites) that rapidly eliminate replicating cells, including not only tumor cells but also normal tissue cells, with a broad range of side effects that significantly limit their applications in cancer therapy. In contrast to conventional systemic chemotherapy, molecularly targeted cancer therapies, using novel drugs aimed at the inhibition of intracellular signaling pathways fundamental for cancer proliferation or differentiation (e.g., monoclonal antibodies and low molecular weight protein-kinase inhibitors), are thought to be cancer-specific, with fewer associated adverse effects on normal cells [8]. Lastly, immunotherapies exploit the immune system to enhance antitumor immunity with promising results in certain cancer treatments, by employing immune checkpoint inhibitors (ICIs), chimeric antigen receptor (CAR) T-cell therapy, or the patient’s own T-cells engineered to specifically target cancer cells [9].

Treatment for cancer diseases can adversely affect the heart and the vasculature. Chemotherapy-induced cardiotoxicity manifests as a broad spectrum of cardiac dysfunctions, with heart failure (HF) representing the most severe consequence. It may be acute and transient, when it occurs during or soon after treatment, or chronic, categorized into type I (early onset) and type II (late onset), based on distinct pathological changes and clinical characteristics [10].

Type I cardiotoxicity is dose-related, associated with largely irreversible myocardial ultrastructural changes (e.g., vacuole formation, contractile element disarray, necrosis), leading to left ventricular dysfunction (LVD) or HF [11,12] and is usually caused by anthracyclines and traditional chemotherapeutic agents. Surprisingly, cardiotoxic-related adverse effects may be associated not only with conventional chemotherapy and radiotherapy, but also with novel targeted chemotherapeutic agents and immune-based therapeutic modalities, since they block pathways that are major modulators of myocardial function, especially under conditions of cardiac stress, such as hypertension or hypertrophy [13].

In this regard, type II cardiotoxicity is typically caused by novel biological-targeted antibodies, and it largely differs from type I for both mechanisms and clinical manifestations [14]. Specifically, two types of mechanisms can be identified in type II cardiotoxicity: the “on target” toxicity, associated with specific mechanisms of action of the drug, and the “off target” toxicity, where the indirect or direct inhibition of other signaling pathways by the drug cause symptoms of cardiotoxicity [15]. In general, type II cardiotoxicity is not dose-related, does not show apparent ultrastructural abnormalities, and is characterized by reversible cardiac functional changes and high likelihood of recovery [16].

Cardiac complications represent unresolved and potentially life-threatening conditions in cancer survivors, thus compromising some favorable benefits of modern cancer treatments and, consequently, representing a challenge for clinicians and patients [17]. Early detection and prevention of cardiotoxicity, as well as understanding of the multifactorial interaction among the selected chemotherapeutic regimen, traditional cardiovascular risk factors and individual susceptibility are urgently needed to optimize treatment options and reduce cardiotoxicity [18]. In response to ongoing clinical challenges, recently, personalized medicine and the new interdisciplinary area of cardio-oncology, focusing on the diagnosis, prevention, and management of cardiovascular complications associated with the treatment of malignancy, have been useful in developing new safer therapeutic strategies [17].

### 2.1. Anthracyclines (ANTs)

Anthracyclines (ANTs) [doxorubicin (DOXO), epirubicin, and daunorubicin] are highly effective chemo-therapeutic agents used in the management of hematological and solid tumors, including breast cancer, lymphoma, leukemia, and sarcomas [19], but also in some skin tumors, such as large B-cell lymphoma [20] and cutaneous squamous cell carcinoma [21].

However, these drugs have been recognized as cardiotoxic since the 1960s, particularly in long-term cancer survivors [22].

The cardiotoxic effects result from their antitumor mechanisms and, therefore, effective therapies for cancer treatment-induced cardiotoxicity should affect only cardiotoxic mechanisms without disrupting antitumor pathways. ANT-mediated cardiovascular toxicities include vasospastic and thromboembolic ischemia, hypertension, dysrhythmia, myocarditis, and left ventricular (LV) dysfunction, leading to HF [13].

ANT-related cardiotoxicity represents a significant clinical burden and limits the usability of these drugs, as reported in a recent large prospective study [23]. Interestingly, ANT-induced cardiac damages also affect the right ventricle [24] and the survival of resident progenitor cells [25,26].

ANT-induced cardiotoxicity has not been fully elucidated yet, even though recent studies have proposed new insights on the molecular mechanisms involved [27,28]. The commonly accepted explanation for these cardiotoxic effects is represented by oxidative stress. Specifically, ANT metabolism generates high levels of reactive oxygen species (ROS) and reactive nitrogen species (RNS) that are not cleared by antioxidant enzymes and cause DNA damage and membrane lipid peroxidation, leading to cardiomyocyte death and replacement by fibrous tissue [29].

Mitochondria, which are rich in cardiomyocytes, are the major subcellular target in ANT-induced cardiac damage due to the presence, in these organelles, of cardiolipin and iron. Cardiolipin, a mitochondrial membrane phospholipid involved in apoptotic pathways, interacts with ANTs, resulting in their accumulation in mitochondria where they stimulate ROS/RNS production [30]. In particular, ANTs chelate free iron mostly accumulated in cardiac mitochondria and form complexes that react with oxygen and trigger ROS production and lipid peroxidation [31]. Therefore, ROS and RNS can cause mitochondrial functional damage, energy imbalances, and ultimately cardiomyocyte death.

Nevertheless, several studies demonstrated a lack of therapeutic benefits following anti-oxidant or iron chelator treatments in preclinical models and clinical trials [32,33].

An alternative mechanism for ANT cardiotoxicity is represented by their interaction with the enzyme topoisomerase IIβ (TopIIβ), which is active in cardiomyocytes and it is not required for cell division, whereas TopIIα is highly expressed in cancer cells and it is a target for the antitumor effect of ANTs [28,34,35]. ANTs, by binding TopIIβ, cause continuous DNA double-strand breaks and apoptosis through the activation of the p53 pathway. Cardiomyocyte-specific deletion of TopIIβ protects mice from ANT-induced damage [28].

### 2.2. ErbB2 Inhibitors

The HerbB2 family is involved in the stimulation of tumor growth and survival [36]. Inhibitors of HerbB2 are mainly used for the treatment of human breast cancers because of the strong overexpression of this factor in this type of tumors [37]. Trastuzumab is a humanized monoclonal antibody directed against HerbB2 and was the first to be clinically used. Nevertheless, patients treated with this antibody were found to be at increased risk of cardiotoxicity represented by the development of LV systolic dysfunction and clinical HF [38]. Additionally, concomitant administration of trastuzumab and ANTs showed additive adverse cardiac effects [39].

The cardiotoxicity of these inhibitors is related to their capacity to disrupt the protective ErbB2 pathway activated in cardiomyocytes by the growth factor neuregulin-1 (NRG-1) in response to stress [40]. Since this pathway is critically involved also in ANT-mediated cardiotoxicity, its inhibition by ErbB2 inhibitors would explain the enhanced cardiotoxicity in the presence of a combination of ANTs and anti-ErbB2 monoclonal antibodies.

### 2.3. VEGF Inhibitors and Multi-Targeted Kinase Inhibitors

The VEGF signaling pathway inhibitors can target VEGF or the extracellular or intracellular domain of its receptor. At present, four major classes of VEGF inhibitors (VEGFi) are currently used in the clinic, including monoclonal antibodies against VEGF or its receptor, soluble decoy receptors, and small molecules that inhibit the tyrosine kinases (TKIs). Bevacizumab, a humanized monoclonal antibody directed against all isoforms of VEGF, was the first angiogenesis inhibitor to be approved by the FDA in 2004 for the treatment of metastatic colorectal cancer. In the following years, its use has been extended to advanced nonsquamous non-small cell lung cancer (NSCLC), renal cell carcinoma (RCC), ovarian cancer, glioblastoma multiforme, advanced cervical cancer, and skin tumors such as basal cell carcinoma [41]. The use of bevacizumab can induce cardiac dysfunction in 1–3% of patients [14]. This drug can induce severe hypertension that persists upon discontinuation of the treatment and was associated with a 2.1 fold increase in the risk of cardiac ischemia and arterial thromboembolic events [42,43]. Bevacizumab treatment can also induce arterial thromboembolic events (ATE), especially in older patients, in patients contemporaneously treated with other chemotherapeutic agents, and in those who have experienced previous thrombotic events [43].

Among the small molecule TKIs used as anti-angiogenic agents, sunitinib and sorafenib were the first to be approved by the FDA. Sunitinib is used in the treatment of advanced renal cell carcinoma, gastrointestinal stromal tumor (GIST), and pancreatic neuroendocrine tumor and is associated with the development of hypertension with an incidence ranging from 5% to 47% in different studies [14] and of arterial and venous thrombosis events [44]. Sorafenib is approved for the treatment of unresectable hepatocellular carcinoma and advanced renal cell carcinoma. Its use is associated with an increase in the risk of hypertension. Two meta-analyses based on prospective clinical trials in various types of malignancies revealed a relative risk (RR) of 3.07, 95% CI, 2.05–4.60, *p* < 0.01) and an overall incidence of 19.1% [45]. Sorafenib can also induce an increase in the risk of ATE (1.7%) [44], and, in almost 40.5% of patients treated with this drug, a prolongation of the QT/QTc interval has been observed that can lead to increased risk of ventricular arrhythmias [14]. Recently the approved TKIs, regorafenib, pazobanib, and axitinib induced similar cardiotoxic effects [14].

The pathophysiological mechanisms of cardiotoxicity related to these TKIs are associated with the inhibition of non-specific targets. TKs, indeed, although developed to selectively inhibit VEGF receptor, show activity on structurally unrelated tyrosine kinase receptors. For instance, the inhibition of platelet-derived growth factor receptor (PDGFR), impairing the growth and survival of pericytes, affects cell survival and cardiac adaptation to afterload stress [46].

### 2.4. Anti-BCR-abl Agents

The strategy to target TKs has revolutionized the treatment and outcome of patients affected by chronic myeloid leukemia (CML), a myeloproliferative disorder characterized by a chromosomal translocation that leads to the formation of the BCR–ABL1 fusion gene and to the constitutive activation of the ABL tyrosine kinase [47]. In 2001, imatinib was the first TKi approved for the treatment of CML [48]; however, due to development of resistance to this drug in some patients, second generation (dasatinib, nitolinib, bosutinib) and third generation (posatinib) TKi have been developed. These drugs differ in their potency and activity against BCR–ABL1 and other kinases, explaining their diverse cardiotoxic effects. Indeed, although at the beginning of their use in the clinic all five drugs appeared safe for the heart, subsequent observations reported some adverse side effects [49].

Several studies showed an excellent cardiovascular safety record for imatinib, although in 2006 Kerkela et al. reported a case series of 10 patients along with in vitro and murine studies suggesting that this drug could induce severe cardiac dysfunction and HF [50]. The mechanism proposed for imatinib-induced adverse effects was related to the alteration in the endoplasmic reticulum and mitochondrial homeostasis, with consequences for apoptotic response and protein import in the mitochondrial matrix of cardiomyocytes [51]. Interestingly, some studies suggest even a cardio protective role for imatinib by reduction of the endothelial barrier dysfunction and lowering of the blood glucose level, thus preventing the development of atherosclerotic lesions. By targeting the PDGFR pathway, imatinib improved hemodynamics in patients with advanced pulmonary arterial hypertension (PAH) and attenuated myocardial remodeling in rats [14].

Nilotinib is an orally bioavailable drug used in CML patients resistant to previous therapies. Although during the very first clinical study, no relevant vascular adverse effects were observed, several other clinical studies over the last 5 years have demonstrated an increased risk of peripheral artery disease (PAD) [52]. This adverse event has been related to the metabolic effect of nilotinib and to its influence on the endothelium, platelets, and on the coagulation process [14,52].

Dasanitib is classified as a dual Abl/Src inhibitor, but it is active on a broad spectrum of receptor kinases. The cardiotoxicity of this drug is similar to that of imatinib with the addition of pleural effusion considered, in part, the result of PDGFR inhibition. PAH is observed in a small percentage of patients (2.4–5%) and in most cases is completely or partially reversible [14]. The mechanism behind this adverse effect is still poorly understood, but it has been suggested that it could be related to the inhibition of Src kinases [53].

Bosutinib is a second generation dual Src and ABL TKI with minimal activity against PDGFR or c-KIT [54]. During long-term bosutinib therapy, the cardiovascular (CV) events were rare, and, in most of the cases, the patients did not need to interrupt the treatment.

Ponatinib is the only third generation BCR–ABL TKi available and is characterized by its ability to inhibit a broad spectrum of TK receptors. Its use is accompanied by an increased risk of arterial thrombotic events (cardiac, cerebral, and peripheral) but the mechanisms related to these cardiotoxic effects are still not well known [55].

### 2.5. Immunotherapy and Radiotherapy

Cancer immunotherapy is a newly emerging treatment method [56], and in particular, checkpoint inhibitors have shown very promising results in different solid and hematological cancers and in skin tumors, such as cutaneous malignant melanomas, Merkel cell carcinoma, basal cell carcinomas, squamous cell carcinoma, and Kaposi Sarcomas [57]. However, the use of this kind of inhibitors in the clinic is associated with a spectrum of adverse events that are known as immune related adverse events (IRAEs). Although monoclonal antibodies targeting programmed cell death 1 (PD-1) or programmed death-ligand 1 (PD-L1) proteins have shown very low toxicity, some cases of myocarditis have been reported after nivolumab or pembrolizumab (anti PD-1 inhibitors). However, it should be noted that PD-1 has an important role in cardiac homeostasis and response to stress; therefore, caution should be taken using these inhibitors.

Radiation therapy (RT) is a clinical treatment focused on the use of ionizing radiation, which has the goal of destroying different forms of neoplasia [58] and skin cancers, such as basal cell carcinoma and squamous cell carcinoma [59].

The two main electromagnetic radiations used are X-rays and gamma (γ) rays. At high doses, these rays kill cancer cells or slow their growth by damaging their DNA [60,61].

Although the techniques of radiotherapy have improved in recent years, in most cases, the heart receives high radiation doses that cause radiation-induced heart disease (RIHD) with harmful consequences for the patients [62].

Radiation therapy can exert cardiotoxic effects in cardiac ECs, rather than cardiomyocytes, because of the post mitotic state of these latter cells. Specifically, radiation increases oxidative stress, resulting in the up-regulation of ROS and inflammation, which in turn decrease perfusion and cause myocardial ischemia [63].

### 2.6. Other Antineoplastic Drugs

Other cancer therapies that induce cardiotoxicity are represented by taxanes, antimetabolites, and proteasome inhibitors [14].

Taxane-mediated cardiotoxicity could be associated with myocardial damage via effects on subcellular organelles [64] or to massive histamine release, resulting in conduction disturbances and arrhythmias [65]. Antimetabolites mainly induce vascular endothelial damage [66], while proteasome inhibitors have dangerous effects mainly on cardiomyocytes but also on ECs by altering the protein synthesis–degradation balance [67]. Taxanes, antimetabolites and proteasome inhibitors, as anti-ErbB2 inhibitors, enhance cardiotoxicity in combination with ANTs by inducing the formation of toxic ANT metabolites [68,69,70].

## 3. Monitoring of Cardiotoxicity

The assessment of anticancer therapy-induced cardiotoxicity before irreversible damage has occurred is crucial. Echocardiography represents a commonly used technique to define sub-clinical cardiotoxicity during and after cancer therapy in survivors as well as equilibrium radionuclide angiography and tissue Doppler imaging [71]. Cardiac magnetic resonance imaging can assess myocardial function more accurately. It is reproducible and reliable but time consuming, costly, and has limited availability [72].

Endomyocardial biopsy is the most sensitive tool to grade the severity of anticancer drug-induced cardiotoxicity [73]. Using electron microscopy, it is possible to detect the loss of myofibrils and vacuolization of the cytoplasm. However, the correlation of biopsy scores (represented by the percentage of cells with typical changes) with LV ejection fraction (LVEF) measured by echocardiography is poor due to the ability of the LV to compensate. Further, this technique is not a routine choice in early monitoring because of its invasiveness.

Recently, serum biomarkers, i.e., troponin I (cTn I), B-type brain natriuretic peptide (BNP), and N-terminal pro-brain natriuretic peptide (NT-proBNP), have been validated for predicting cardiotoxicity during anticancer therapy [74]. In particular, cTnI being released in the circulation upon cardiac necrosis, can detect early chemotherapy-associated cardiotoxicity before significant LVEF changes occur, but lacks specificity. In contrast, BNP and NT-proBNP are considered rapid and accurate indicators of HF caused by antineoplastic drugs, since they are stable and can accumulate to high concentrations. Currently, the assessment of cardiac biomarkers is not applied routinely in patients receiving anticancer drugs. Nevertheless, it is noteworthy that the recent Canadian CV Society guidelines suggested the use of these markers for the detection of early development of LV dysfunction in cancer patients under anticancer therapy [74].

Assessment of several miRNAs, discussed below, have also been proposed for early detection of chemotherapy-associated cardiomyopathy [75].

## 4. Role of MicroRNAs in Anti-Cancer Therapy-Induced Cardiotoxicity

The following miRNAs, summarized in Table 1, are the most relevant ones modulated by therapies for cancer, which have been demonstrated to be involved in cardiac diseases.

### 4.1. miR-200 Family

The miR-200 family (miR-200s) includes five members (miR-200a, miR-200b, miR-200c, miR-141, and miR-429). This miRNA family is deeply involved in the epithelial to mesenchymal transition (EMT) of tumor cells [102]. Therefore, anti-cancer therapies modulate the expression of miR-200s.

In particular, it has been shown that the expression levels of miR-200c are induced by doxorubicin (DOX) in cardiac mesenchymal progenitor cells (CmPC) [103]. Oxidative stress and DNA damage response are considered the main mechanisms involved in DOXO-mediated cardiotoxicity [104,105]. miR-200c is an oxidative stress-induced miRNA that has been linked to endothelial dysfunction, since it induces apoptosis and senescence in ECs via the downregulation of ZEB1 protein [106] and induces NO decrease and oxidative stress increase downregulating Sirtuin1 (SIRT1), endothelial nitric oxide synthase (eNOS), and Forkhead boxO1 (FOXO1) [107], three proteins that regulate EC homeostasis [108]. In a mouse model of cardiotoxicity, it has been shown that Stromal cell-derived factor 1 (SDF1) administration partially reverted DOXO-induced miR-200c and p53 protein upregulation in mouse hearts [103]. In addition, the demise of ZEB1 mRNA and protein induced by DOXO was significantly prevented by SDF1. In keeping, p21 mRNA, which is induced by p53 and inhibited by ZEB1, is induced by DOXO treatment and is decreased by SDF1 administration. Interestingly, SDF1 plays a cardioprotective in DOX-treated mice, partially reverting the adverse remodeling, decreasing LV end diastolic volume, LVEF, and LV anterior wall thickness in diastole, recovering LV end systolic pressure and reducing ±dP/dt [103].

On the other hand, it has been shown that miR-200a levels were decreased in DOX-treated mice and in rat cardiomyoblast cell line H9c2 exposed to DOX [109]. The authors show that miR-200a reduced oxidative stress and cardiac apoptosis without affecting matrix metalloproteinase and inflammatory factors in mice with acute DOX injection, since miR-200a targets Kelch like ECH associated protein 1 (Keap1), resulting in nuclear factor erythroid 2-related factor 2 (Nrf2) activation [109].

Although these studies seem to be in contrast, it is clear that miR-200 family member modulation is deeply involved in cardiovascular homeostasis affected by cancer treatments.

### 4.2. miR-34 Family

The miRNA-34 family consists of 3 highly homologous microRNAs, namely miRNA-34a, b, and c. This family has been shown to be modulated by different anti-cancer treatments, such as anthracyclines [76,110,111]. In particular, miR-34a has been shown to be up-regulated in the myocardium and plasma of DOX-treated rats and in rat cardiomyocyte H9c2 cells treated with DOX [76].

Interestingly, dexrazoxane (DEX), a treatment that is known to prevent anthracycline-induced cardiomyopathy [112], was able to reverse miR-34a increase in rats treated with DOX [76].

Human miR-34a was also shown to be increased in the plasma of patients with diffuse large B-cell lymphoma after 9 and 16 weeks of epirubicin therapy [76].

In H9c2, miR-34a was shown to induce BCL2 associated X, apoptosis regulator (Bax) and to inhibit B-cell lymphoma 2 (Bcl-2) expression, activating caspase-3 and mitochondrial potentials. Moreover, miR-34a targets SIRT1, which is known to deacetylate p66ShcA gene promoter [113]. Therefore, miR-34a-dependent SIRT1 demise enhances p66shc protein increase, which is a redox enzyme implicated in mitochondrial ROS generation and in the translation of oxidative signals [114].

Through this mechanism, miR-34a, by targeting the Sirt1/p66shc pathway, contributes to DOX-induced cardiotoxicity [76].

miR-34b/c has also been shown to be upregulated in DOX-treated murine adult cardiomyocyte cell line HL-1 [111]. The authors demonstrated that itchy E3 ubiquitin protein ligase (ITCH) is a direct target of miR-34b/c and that miR-34b/c decreased HL-1 viability, promoting NF-kB expression and increasing proinflammatory cytokines, such as TNF-a and IL-6, via ITCH downmodulation. In keeping, miR-34 antagomir protected myocardial cells in a mouse model of cardiomyopathy [111].

All these studies show that the entire miR-34 family plays a major role in anthracycline-induced cardiotoxicity.

miR-34a was also shown to be up-regulated in human cardiomyocytes exposed to radiation [77]. miR-34a is of great interest in radiobiology, since it plays different roles in radiation response. Consequently, is a potential therapeutic target in tumor radio resistance and in tissue radiotoxicity. Moreover, its expression is under the control of p53 oncoprotein, which is induced by ionizing radiation [78].

Moreover, as previously described, miR-34a modulates ROS and inflammation production; in keeping, the migration inhibitory factor (MIF) cardio-protective cytokine was shown to decrease miR-34a levels in human cardiomyocytes exposed to ionizing radiation, reducing the radiation-associated senescence through the up-regulation of the miR-34a protein target SIRT1 [77].

miR-34a plays an important role as an immunotherapeutic agent too, since it targets PD-L1, a target of monoclonal antibodies used for immunotherapy already described. PD-L1 suppression can cause autoimmune myocarditis and deletion of PD-1 in mice, causing dilated cardiomyopathy, impaired contraction, and heart failure [80,97,115,116].

Therefore, miR-34a modulation can be exploited as an immunotherapy strategy; indeed, a liposomal formulation of miR-34a (MIRX34) is currently in a phase I clinical trial [117].

In vivo, MIRX34 increased the number of tumor infiltrating CD8+ T-cells and decreased the number of exhausted CD8+PD1+ T-cells and macrophages, suggesting that miR-34 may have a direct effect on immune evasion that can be exploited therapeutically. In combination with radiotherapy, the effect on CD8+ T-cells was improved, and it has also been shown to induce adaptive immune responses [79].

The efficacy of miR-34a to modulate the antitumor immune responses and control tumor growth in combination with radiotherapy was demonstrated. The ability of miR-34a to potently control immune responses was proven by the occurrence of five immune-related serious adverse events, which determined the early termination of the phase I clinical trial (on September 2016). Further in-depth studies on toxicity and immune-related adverse events were conducted in patients with advanced solid tumors.

Moreover, given the detrimental role of miR-34a in cardiotoxicity, as described above, miR-34a in vivo delivery should be carefully evaluated.

### 4.3. miR-29 Family

The miR-29 family is composed of different miRNAs, namely miR-29a, miR-29b, and miR-29c, which share a common seed sequence and differ for 2 to 3 bases.

It was shown that this family is modulated by different anti-cancer treatments.

miR-29b is the member of the miR-29 family that was significantly downregulated in myocardium of DOX-treated rats [118]. Rescue of miR-29b expression in the myocardium resulted in a marked improvement of cardiac function. miR-29b overexpression in rat cardiomyocytes decreased DOX-induced cardiomyocyte apoptosis, since miR-29b targets directly the anti-apoptotic protein Bax [118].

In a different study an increase was shown of miR-29b in the plasma of children or young adults treated with anthracycline chemotherapy (AC). Plasma miR-29b expression was elevated post-AC, and a dose response relationship with anthracycline dose and markers of cardiac injury was observed [119].

miR-29 family members are inhibitors of cardiac fibrosis and play a major role in cardiac remodeling following cardiomyocyte injury [120]. Indeed, miR-29a upregulation following myocardial injury has been reported, and the degree of mR-29a upregulation was associated with the extent of late remodeling post-acute myocardial infarction [121]; in addition, higher levels of miR-29a have been found in the plasma of patients with cardiac hypertrophy and are inversely associated with cardiac fibrosis [122].

miR-29b targets different genes involved in the extracellular matrix (ECM), such as fibronectin, collagen, and matrix metalloproteinases [123]. Since early and late ECM remodeling plays a major role in response to AC-induced cardiotoxicity [81,124], miR-29 up-regulation may reflect early remodeling in response to AC-induced cardiac injury.

miR-29b was also shown to be downregulated in irradiated vs. non irradiated arteries from patients receiving microvascular free tissue transfer reconstructions. Moreover, in ApoE–/– mice receiving a single irradiation dose in a designated mediastinal and neck area, including the heart and large vessels, miR-29b was downregulated in irradiated arteries [82]. miR-29b targets pentraxin-3 and dipeptidyl-peptidase 4, which regulate inflammatory and matrix protein binding; therefore, a reduction of miR-29b could increase the vascular inflammatory response.

In a different study, circulating miR-29a levels were found to be decreased in plasma samples of patients with non-small cell lung cancer (NSCLC) after radical thoracic radiotherapy. The decrease of miR-29a levels were related to RT dose used [125].

Thus miR-29 downregulation by radiotherapy could predict a negative impact on vascular inflammation.

### 4.4. miR-30 Family

The miR-30 family consists of five members (miR-30a, miR-30b, miR-30c, miR-30d, and miR-30e). This miRNA family was found downregulated by DOX in cardiomyocytes and in the heart of rats [126,127]. The decrease of miR-30 has been shown to be cardioprotective; in fact, miR-30 expression attenuated the contractile response of cardiomyocytes to β-adrenoceptor (βAR) stimulation. Moreover, miR-30 expression increased cardiac cell viability upon DOX treatment [128]. In keeping, GATA6 (a transcription factor known to play a key role in cardiac development) inhibits miR-30 transcription. GATA6 is induced by DOX triggering miR-30 downregulation [128].

In NSCLC patients treated with bevacizumab chemotherapy, serum miR-30c levels were detected at pre-chemotherapy, during-chemotherapy, and after chemotherapy. miR-30c expression was found correlated with duration of the chemotherapy cycle and decreased 1 month after chemotherapy. Moreover, correlation analysis showed that serum miR-30c levels were positively related to cardiotoxicity before chemotherapy and during chemotherapy [129].

### 4.5. miR-21

miR-21 has been shown to be modulated by several anticancer treatments described below and seems to play both positive and negative functions in cardiotoxicity.

miR-21 has been shown to be up-regulated in the myocardium of chronically DOX-treated mice, whereas it was not modulated under acute DOX treatment [130]. The increase of miR-21 was also observed in vitro in H9C2 cells exposed to different concentrations of DOX [130]. miR-21 has an anti-apoptotic function in ischemia-induced cardiomyocyte death mediated by the direct inhibition of the pro-apoptotic targets, such as programmed cell death 4 and activator protein-1, inducing different mediators of cardioprotection including eNOS, heat shock protein 70, and heat shock transcription factor-1 [83,84]. Moreover, miR-21 anti-apoptotic effects are also achieved through its inhibition of B cell translocation gene 2 (BTG2), a gene involved in cell proliferation, DNA damage repair, differentiation, and apoptosis in cancer cells [130].

On the other hand, miR-21 is involved in fibrosis and remodeling, since it targets phosphatase and tensin homologue (PTEN) expression [85]. The inhibition of PTEN causes matrix metalloprotease-2 (MMP2) increase, contributing to cardiac remodeling. In keeping with these findings, miR-21 levels are also selectively increased in the failing heart fibroblasts, up-regulating ERK-MAP kinase activity through Sprouty homologue 1 inhibition [86]. Thus miR-21 regulates fibroblast survival and growth factor secretion, controlling interstitial fibrosis; it is highly expressed in cardiac fibroblasts in mice, and miR-21 knockdown was able to regress cardiac fibrosis and hypertrophy in mice [86].

miR-21 plays also a pivotal role in radiation-induced toxicity. Its expression also in this case was shown to be induced by ionizing radiation in human fibroblasts [131] and in the myocardium of rats exposed to chest irradiation. miR-21 up-regulation was shown to modulate extracellular matrix proteins and PKC signaling, which may affect electrical coupling mediated by connexin 43 (Cx43) [87]. Moreover, in peripheral blood mononuclear cells (PBMCs), high miR-21 levels were detected after radiotherapy in association with acute genitourinary radiotoxicity [88].

miR-21 is another miRNA whose modulation could play an important role as an immunotherapeutic agent, since it is deeply involved in PD-L1 expression.

Tumor cells, in fact, promote the expression of miR-21 in macrophages, which inhibit STAT1, JAK 2, and the activation of NF-κB, preventing the anti-tumoral M1 polarization. In keeping, genetic deficiency of miR-21 drives tumoricidal M1 polarization and confers an anti-tumor immunity [132].

Moreover, PD-L1 expression is regulated by IFN-γ-mediated STAT1 activation [80] and is upregulated by miR-21 depletion and the consequent STAT1 activation in cultured bone marrow-derived macrophages and in tumor-associated macrophages (TAM) residing in tumors.

PD-1 antibodies and miR-21-deficient macrophages act synergistically as anti-tumor therapy with an activity superior to either agent alone. In conclusion, miR-21 depletion enhances the host immune system against tumor development through M1 polarization of TAMs [132].

Since an increase of miR-21 seems to play a detrimental role in cardiac tissue, the miR-21 inhibition strategy could also ameliorate most of the cardiotoxic effects provoked by different anticancer strategies.

### 4.6. MyomiRs

A subset of miRNAs plays an important role in survival and proliferation and muscle differentiation; which is known as MyomiR, i.e., muscle specific miRNA [133]. These include miR-1 and miR-133a/b, miR-499, and miR-208a/b. Since MyomiRs play a fundamental role in heart homeostasis, they have been studied in many cancer treatments that induce cardiotoxicity.

#### 4.6.1. miR-1

miR-1 is a skeletal muscle specific miRNA that plays a pivotal role in cardiomyocyte differentiation and which has an antiproliferative effect. [134,135]. miR-1 is up-regulated in response to ischemia/reperfusion (I/R) injury in rat heart and in a rat model of myocardial infarction [89,90]. Moreover, miR-1 is upregulated in the heart of patients with myocardial infarction (MI) [91].

Serum levels of miR-1 were up-regulated in acute myocardial infarction (AMI) in rats and humans. miR-1 levels showed a strong positive correlation with MI size in rats [136] and in humans positively correlated with serum creatine kinase– myocardial band (CK–MB) levels [136].

Circulating miR-1 was found up-regulated in DOX-treated rats and in breast cancer patients treated with DOX [137,138].

miR-1 levels were associated with changes in LVEF, and its levels were useful to discriminate patients affected by cardiotoxicity from unaffected subjects better than cTnI levels [138].

In irradiated rats, miR-1 was found downregulated in the heart [87,139].

miR-1 plays a pivotal role in electrical coupling and direct cardiac cell to cell communication to ensure heart function, since it targets intercellular Cx43 channels [140].

In rats irradiated with a single ionizing radiation, miR-1 was found decreased, and a concomitant Cx43 increase was observed causing myocardial intercellular communication enhancement, resulting in a beneficial heart response [87].

In conclusion, circulating miR-1 modulation seems to reflect anthracycline toxicity; on the other hand, irradiation-induced downregulation in the heart plays a beneficial effect.

#### 4.6.2. miR-133

miR-133 are two miRNAs, namely miR-133a and miR-133b, which share the same seed sequence and are muscle specific miRNAs highly expressed in human heart [141].

miR-133a/b have been demonstrated to be involved in cardiac hypertrophy; indeed, an miR-133 decrease positively regulates cardiac hypertrophy, increasing the expression of its targets, including calcineurin, NFATc4 (regulator of hypertrophy), Rac, and Cdc42 (regulators of cardiac prohypertrophic mitogen-activated protein (MAP) kinase pathway [84].

Moreover miR-133a/b has an anti-apoptotic effect since it inhibits caspase-9 expression [142].

miR-133a and miR-133b have been shown to increase in the plasma of rats treated with DOX to induce cardiotoxicity; albeit an appreciable variation of expression associated with cardiotoxicity onset was not found [143].

#### 4.6.3. miR-208a/b

miR-208a/b are embedded within the introns of myosin genes: miR-208a into α-MHC (also known as *Myh6*) and miR-208b into β-MHC (*Myh7*).

In the adult mouse heart, alpha-MHC/miR-208a dominates, whereas miR-208b is exclusive for the healthy human heart.

miR-208 is involved in the regulation of the myosin heavy chain (MHC) isoform switch during development and in pathophysiological conditions in mice.

In DOX-treated mice, miR-208a is increased in the hearts and induces cardiomyocytes apoptosis; moreover, therapeutic silencing of miR-208a increased its protein targets GATA4, which is a transcription factor known to regulate the expression the antiapoptotic gene Bcl-2; therefore, miR-208a downregulation is able to counteract myocyte apoptosis in DOX-treated animals [92].

Moreover, antagomiR-208a treatment improved also cardiac function assessed by cardiac imaging [92].

Circulating levels of miR-208a were shown to be increased in a rat model of cardiotoxicity induced by DOX, suggesting its role as a plasma biomarker for cardiotoxicity in rats [137].

Notwithstanding this, circulating levels of miR-208a were not found detectable in doxorubicin-induced cardiotoxicity in breast cancer patients [93].

In a different study, the expression levels of miR-208a in rat hearts decreased during the DOX treatment (cumulative doses), similarly with its encoding geneMyh6, whereas miR-208b levels were increased [110].

miR-208b was found up-regulated also in heart of mice treated with DOX [94], and studies on circulating miR-208b described its modulation.

#### 4.6.4. miR-499

miR-499 is another myomiR embedded *in* β-MHC (*Myh7b*) genes modulated by chemotherapy.

Indeed, it is up-regulated in plasma of children and young adults treated with anthracyclines, and the expression significantly correlated with AC dose. Patients with acute cardiomyocyte injury demonstrated higher expression of miR-499 post-AC compared with those without [119]. On the other hand, miR-499 was significantly downregulated in DOX-treated mice heart, while the serum miR-499 expression was significantly increased [144].

It has been shown that miR-499 targets p21, and p21 downregulation significantly decreased mitochondrial fission and cell death in cardiomyocytes exposed to DOX. Therefore, upon DOX administration, the decrease of miR-499 induced abnormal mitochondrial fission and cell apoptosis in the mouse heart [144].

### 4.7. miR-221/222

miR-221/222 are highly homologous miRNAs that share the same seed sequence that are encoded in tandem on the X chromosome in human, mouse, and rat and are highly conserved in vertebrates. They play a key role in the development of cancer, acting either as oncomiR or as oncosuppressor [145].

In addition, these two miRNAs are highly expressed in vascular smooth muscle cells (VSMCs) and ECs, and they have been extensively studied in vascular cell physiology [146].

In particular, miR-221/222 have pro-migration, pro-proliferative, and anti-apoptotic effects in VSMCs, whereas they have antiproliferative, anti-migration, and pro-apoptotic effects in ECs [146].

Reduced myocardial miR-221/222 expression is associated with severe cardiac fibrosis in heart failure patients [95]. miR-221 overexpression has been shown to induce cardiac hyperthropy in vitro [96] and to promote HF [147]. Indeed, miR-221/222 are significantly upregulated in patients with hypertrophic cardiomyopathy [95].

Notably, circulating serum miR-221 levels are lower in patients with HF than in healthy controls [98].

In a mouse model of cardiotoxicity induced by DOX, miR-221/222 were found up-regulated in the heart; moreover, radiotherapy also induced and up-regulation of miR-221/222 in the blood of breast cancer patients treated with radiotherapy, and the levels of miR-221/222 were affected by cardiovascular disease [94,99].

Therefore, miR-221/222 modulation by anticancer treatments seems to be deeply involved in cardiotoxicity induction.

### 4.8. miR-320a

The microRNA miR-320 family consists of five members, namely miR-320a, -b, -c, -d, and -e. miR-320 term is used most frequently, but the most studied member is miR-320a [148].

miR-320 has been shown to regulate physiological processes such as cardiac survival (apoptosis) [149] and glucose-induced gene expression in diabetes [150].

miR-320a is increased in cardiomyocytes and ECs after DOX-treatment, and it is involved in DOX-induced cardiotoxicity since it targets directly VEGF-A [151]. Therefore miR-302a upregulation and decreasing VEGF-A alters cardiac vascular homeostasis.

Moreover, miR-320a inhibition attenuates DOX-induced cell growth arrest and apoptosis, while its overexpression worsens these effects. Additionally, miR-320a overexpression impairs NO release, tube formation, and EC cell migration. In vivo miR-320a inhibition reduced cardiac abnormalities provoked by DOX. On the contrary, overexpression of miR-320a enhanced apoptosis in vitro and provoked vessel abnormalities in the heart and cardiac dysfunction in mice [151].

Furthermore, miR-320a can target other molecules involved in angiogenesis regulation, such as insulin-like growth factor (IGF), IGF receptor (IGFR), and neuropilin-1 I (NRP1). The IGF1–IGFR pathway has been proved to have protective effects on DOX-induced cardiotoxicity [152,153], and NRP1 is a co-receptor for VEGF-A [100].

Circulating miR-320a levels were found downregulated in five DOX-treated acute myeloid leukemia (AML) subjects compared to five control donors [151].

All these studies suggest that miR-320a plays important roles in DOX-induced cardiotoxicity, although further studies are necessary to elucidate possible therapeutic options.

## 5. Treatment of Cardiotoxicity

There are several other methods to prevent anticancer-induced cardiac damage. Unlike ErbB2 inhibitors, the total cumulative dose of ANTs is one of the most significant risk factor for cardiac dysfunction. Therefore, prolonging infusion duration rather than administering a bolus dose can prevent and/or reduce cardiotoxicity in patients that have to receive high doses of ANTs [154]. The use of liposome-encapsulated ANTs can also reduce the accumulation of these drugs in the heart, since their presence is restricted to the intravascular space. Therefore, liposomal ANTs do not accumulate in the heart, while they selectively enter the tumor tissue characterized by vascular endothelial discontinuity and breakage [155].

Other approaches to counteract chemotherapy-associated cardiotoxicity include different pharmacologic interventions and also nutritional supplementation and exercise training [101,156,157]. Considering ANT treatments, since the main mechanism of ANT cardiotoxicity is represented by oxidative stress, the use of antioxidants seems the most promising cardioprotective strategy. Among them, DEX, first studied in beagles in the early 1980s [158], exerts a significant cardioprotective effect in cancer patients under ANT therapy without affecting the antitumor efficacy [104]. Acting as an iron chelating agent, it interferes with mitochondrial iron-mediated ROS production. Nevertheless, its cardioprotective effect does not stem only from its antioxidant properties, since it has been shown that DEX also prevent the interaction of ANTs with TopIIβ and, therefore, DNA double-strand breaks without lowering ANT’s anticancer effects [159]. Currently, DEX is the only cardioprotective drug approved for clinical use by the Food and Drug Administration for ANT cardiotoxic affects. B,β-blockers with antioxidant properties, such as carvedilol and nebivolol, have also shown promising results as cardioprotective agents [160,161]. Nevertheless, data from large randomized clinical trials demonstrating the beneficial effects of these drugs for the prevention of cardiotoxicity under contemporary ANT therapy are still limited.

A cardioprotective effect has been also observed with the use of angiotensin-converting enzyme inhibitors (ACEI), and it is mainly based on the ability of these drugs to attenuate oxidative stress but also reduce interstitial fibrosis and avoid intracellular calcium overload. Nevertheless, a combined therapy with ACEI and β-blockers appears to be more beneficial than an ACEI monotherapy, as demonstrated by recent clinical trials [162]. Other promising drugs tested to counteract ANT cardiotoxicity are represented by statins, phosphodiesterase-5-inhibitors, and ranolazine [29]. Whether ACEI exerts beneficial effects in preventing ErbB2 cardiotoxicity still remains to be elucidated. Nevertheless, data from a very recent randomized trial suggested that in patients with breast cancer treated with trastuzumab, both ACEI or β-blockers reduced trastuzumab-induced cardiotoxicity [163].

It has been hypothesized that administration of the recombinant protein NRG-1 to cancer patients can be used to improve cardiac chamber dimensions and LV function due to its cardioprotective properties via ErbB4/ErbB2 signaling. Clinical studies confirmed this hypothesis in patients with chronic HF [164,165]. However, concerns have been raised over increased proliferation of tumor cells even though, recently, a bivalent neuregulin has been described that is able to protect against DOX cardiotoxicity without interfering with doxorubicin-mediated antitumor effects [166].

## 6. Conclusions and Future Perspectives

As previously described, different pharmacological strategies are in use to downregulate cardiotoxicity.

Among these, miRNA modulation holds good promise as a therapeutic strategy to counteract cardiotoxicity induced by anticancer treatments. miRNAs, in fact, are useful both as biomarkers of cardiotoxicity and for target therapy, since they modulate entire signaling pathways. Unfortunately, many miRNAs modulated by anticancer treatments are also involved in cardiotoxicity. Therefore, the comprehension of the mechanisms elicited by miRNAs and the amelioration of specific delivery in either cardiac or tumor regions, could help to reduce negative side effects.

Interestingly, it has been shown that treatment with exosomes of cardiac mesenchymal progenitor cells injected systemically in a mouse model of cardiotoxicity obtained with DOX/trastuzumab treatment was able to decrease ROS and inflammation and LV dysfunction. The vesicles were highly enriched in miR-146a compared with human dermal fibroblast exosomes, a miRNA that plays a cardioprotective role [167].

Hence, a miRNA-therapy could be a useful tool for the prevention and cure of cardiotoxic effects of cancer therapies.

## Figures and Tables

**Table 1:** miRNA modulated by anticancer treatments.

miRNA	Cancer Treatment	Modulation	Tissue/Cells	Source	Ref.
miR-200c	DOX	up	hCmPC	human	[76]
	DOX	up	LV heart	mouse	[76]
miR-200a	DOX	down	rat cardiomyocytes	rat	[77]
miR-34a	DOX	up	myocardium, plasma, cardiomyocytes	rat	[78]
	epirubucin	up	plasma	B-cell lymphoma pts	[78]
	IR	up	cardiomyocytes	human	[79]
miR-34 b/c	DOX	up	cardiomyocyte cell line	mouse	[80]
miR-29b	DOX	down	myocardium, cardiomyocytes	rat	[81]
	AC	up	plasma	young cancer pts	[82]
	IR	down	arteries	human	[83]
	IR	down	arteries	ApoE^–/–^ mice	[83]
miR-29a	RT	down	plasma	NSCLC pts	[84]
miR-30 family	DOX	down	cardyomyocytes, heart	rat	[85,86]
miR-30c	bevacizumab	up	serum	NSCLC pts	[87]
miR-21	DOX	up	myocardium	mouse	[88]
	DOX	up	cardiomyocytes	rat	[88]
	IR	up	fibroblasts	human	[89]
	IR	up	myocardium	rat	[90]
	RT	up	PBMCs	Prostate cancer pts	[91]
miR-1	DOX	up	plasma	rat	[92]
	DOX	up	plasma	Breast cancer pts	[93]
	IR	down	myocardium	rat	[90,94]
miR-133a/b	DOX	up	plasma	rat	[95]
miR-208a	DOX	up	myocardium	mice	[96]
	DOX	up	plasma	rat	[96]
	DOX	down	myocardium	rat	[97]
miR-208b	DOX	up	myocardium	rat	[97]
	DOX	up	myocardium	mouse	[98]
miR-499	DOX	down	myocardium	mouse	[99]
	DOX	up	serum	mouse	[99]
miR-221/222	DOX	up	myocardium	mouse	[98]
	RT	up	plasma	Breast cancer patients	[100]
miR-320a	DOX	up	endothelial cells	human	[101]
	DOX	up	cardiomyocytes	rat	[101]
	AC	down	blood	AML patients	[101]

Abbreviations: DOX, doxorubicin; IR, ionizing radiations; RT, radiotherapy; AC anthracycline chemotherapy; Pts, patients; AML, acute myeloid leukemia; NSCLC, non-small cell lung cancer.

## References

[1] Bartel D.P. (2009). MicroRNAs: Target recognition and regulatory functions. Cell.

[2] Huntzinger E., Izaurralde E. (2011). Gene silencing by microRNAs: Contributions of translational repression and mRNA decay. Nat. Rev. Genet..

[3] Winter J., Jung S., Keller S., Gregory R.I., Diederichs S. (2009). Many roads to maturity: microRNA biogenesis pathways and their regulation. Nat. Cell Biol..

[4] Zhu H., Fan G.-C. (2011). Extracellular/circulating microRNAs and their potential role in cardiovascular disease. Am. J. Cardiovasc. Dis..

[5] Olson E.N. (2014). MicroRNAs as therapeutic targets and biomarkers of cardiovascular disease. Sci. Transl. Med..

[6] Eschenhagen T., Force T., Ewer M.S., de Keulenaer G.W., Suter T.M., Anker S.D., Avkiran M., de Azambuja E., Balligand J.-L., Brutsaert D.L. (2011). Cardiovascular side effects of cancer therapies: A position statement from the Heart Failure Association of the European Society of Cardiology. Eur. J. Heart Fail..

[7] Zheng P.-P., Li J., Kros J.M. (2018). Breakthroughs in modern cancer therapy and elusive cardiotoxicity: Critical research-practice gaps, challenges, and insights. Med. Res. Rev..

[8] Sawyers C. (2004). Targeted cancer therapy. Nature.

[9] Dong J., Chen H. (2018). Cardiotoxicity of Anticancer Therapeutics. Front. Cardiovasc. Med..

[10] Jain D., Russell R.R., Schwartz R.G., Panjrath G.S., Aronow W. (2017). Cardiac Complications of Cancer Therapy: Pathophysiology, Identification, Prevention, Treatment, and Future Directions. Curr. Cardiol. Rep..

[11] Billingham M.E., Mason J.W., Bristow M.R., Daniels J.R. (1978). Anthracycline cardiomyopathy monitored by morphologic changes. Cancer Treat. Rep..

[12] Ewer M.S., Ewer S.M. (2010). Cardiotoxicity of anticancer treatments: What the cardiologist needs to know. Nat. Rev. Cardiol..

[13] Suter T.M., Ewer M.S. (2013). Cancer drugs and the heart: Importance and management. Eur. Heart J..

[14] Tocchetti C.G., Cadeddu C., Di Lisi D., Femminò S., Madonna R., Mele D., Monte I., Novo G., Penna C., Pepe A. (2019). From Molecular Mechanisms to Clinical Management of Antineoplastic Drug-Induced Cardiovascular Toxicity: A Translational Overview. Antioxid. Redox Signal..

[15] Force T., Kolaja K.L. (2011). Cardiotoxicity of kinase inhibitors: The prediction and translation of preclinical models to clinical outcomes. Nat. Rev. Drug Discov..

[16] Ewer M.S., Lippman S.M. (2005). Type II chemotherapy-related cardiac dysfunction: Time to recognize a new entity. J. Clin. Oncol..

[17] Han X., Zhou Y., Liu W. (2017). Precision cardio-oncology: Understanding the cardiotoxicity of cancer therapy. NPJ Precis. Oncol..

[18] Chang H.-M., Okwuosa T.M., Scarabelli T., Moudgil R., Yeh E.T.H. (2017). Cardiovascular Complications of Cancer Therapy: Best Practices in Diagnosis, Prevention, and Management: Part 2. J. Am. Coll. Cardiol..

[19] Smith L.A., Cornelius V.R., Plummer C.J., Levitt G., Verrill M., Canney P., Jones A. (2010). Cardiotoxicity of anthracycline agents for the treatment of cancer: Systematic review and meta-analysis of randomised controlled trials. BMC Cancer.

[20] Guyot A., Ortonne N., Valeyrie-Allanore L., Bagot M. (2010). Combined Treatment With Rituximab and Anthracycline-Containing Chemotherapy for Primary Cutaneous Large B-Cell Lymphomas, Leg Type, in Elderly Patients. Arch. Dermatol..

[21] Nakamura K., Okuyama R., Saida T., Uhara H. (2013). Platinum and anthracycline therapy for advanced cutaneous squamous cell carcinoma. Int. J. Clin. Oncol..

[22] Tan C., Tasaka H., Yu K.P., Murphy M.L., Karnofsky D.A. (1967). Daunomycin, an antitumor antibiotic, in the treatment of neoplastic disease. Clinical evaluation with special reference to childhood leukemia. Cancer.

[23] Cardinale D., Colombo A., Bacchiani G., Tedeschi I., Meroni C.A., Veglia F., Civelli M., Lamantia G., Colombo N., Curigliano G. (2015). Early detection of anthracycline cardiotoxicity and improvement with heart failure therapy. Circulation.

[24] Tadic M., Cuspidi C., Hering D., Venneri L., Danylenko O. (2017). The influence of chemotherapy on the right ventricle: Did we forget something?. Clin. Cardiol..

[25] Piegari E., De Angelis A., Cappetta D., Russo R., Esposito G., Costantino S., Graiani G., Frati C., Prezioso L., Berrino L. (2013). Doxorubicin induces senescence and impairs function of human cardiac progenitor cells. Basic Res. Cardiol..

[26] Piegari E., Russo R., Cappetta D., Esposito G., Urbanek K., Dell’Aversana C., Altucci L., Berrino L., Rossi F., De Angelis A. (2016). MicroRNA-34a regulates doxorubicin-induced cardiotoxicity in rat. Oncotarget.

[27] Zamorano J.L., Lancellotti P., Rodriguez Muñoz D., Aboyans V., Asteggiano R., Galderisi M., Habib G., Lenihan D.J., Lip G.Y.H., Lyon A.R. (2016). 2016 ESC Position Paper on cancer treatments and cardiovascular toxicity developed under the auspices of the ESC Committee for Practice Guidelines: The Task Force for cancer treatments and cardiovascular toxicity of the European Society of Cardiology (ESC). Eur. Heart J..

[28] Zhang S., Liu X., Bawa-Khalfe T., Lu L.-S., Lyu Y.L., Liu L.F., Yeh E.T.H. (2012). Identification of the molecular basis of doxorubicin-induced cardiotoxicity. Nat. Med..

[29] Scotti L., Franchi M., Marchesoni A., Corrao G. (2018). Prevalence and incidence of psoriatic arthritis: A systematic review and meta-analysis. Semin. Arthritis Rheum..

[30] Pereira G.C., Pereira S.P., Tavares L.C., Carvalho F.S., Magalhães-Novais S., Barbosa I.A., Santos M.S., Bjork J., Moreno A.J., Wallace K.B. (2016). Cardiac cytochrome c and cardiolipin depletion during anthracycline-induced chronic depression of mitochondrial function. Mitochondrion.

[31] Ichikawa Y., Ghanefar M., Bayeva M., Wu R., Khechaduri A., Naga Prasad S.V., Mutharasan R.K., Naik T.J., Ardehali H. (2014). Cardiotoxicity of doxorubicin is mediated through mitochondrial iron accumulation. J. Clin. Investig..

[32] Dresdale A.R., Barr L.H., Bonow R.O., Mathisen D.J., Myers C.E., Schwartz D.E., D’Angelo T., Rosenberg S.A. (1982). Prospective randomized study of the role of N-acetyl cysteine in reversing doxorubicin-induced cardiomyopathy. Am. J. Clin. Oncol..

[33] Hasinoff B.B., Patel D., Wu X. (2003). The oral iron chelator ICL670A (deferasirox) does not protect myocytes against doxorubicin. Free Radic. Biol. Med..

[34] Lyu Y.L., Kerrigan J.E., Lin C.-P., Azarova A.M., Tsai Y.-C., Ban Y., Liu L.F. (2007). Topoisomerase IIbeta mediated DNA double-strand breaks: Implications in doxorubicin cardiotoxicity and prevention by dexrazoxane. Cancer Res..

[35] Tewey K.M., Rowe T.C., Yang L., Halligan B.D., Liu L.F. (1984). Adriamycin-induced DNA damage mediated by mammalian DNA topoisomerase II. Science.

[36] Slamon D.J., Clark G.M., Wong S.G., Levin W.J., Ullrich A., McGuire W.L. (1987). Human breast cancer: Correlation of relapse and survival with amplification of the HER-2/neu oncogene. Science.

[37] Mitri Z., Constantine T., O’Regan R. (2012). The HER2 Receptor in Breast Cancer: Pathophysiology, Clinical Use, and New Advances in Therapy. Chemother. Res. Pract..

[38] Bowles E.J.A., Wellman R., Feigelson H.S., Onitilo A.A., Freedman A.N., Delate T., Allen L.A., Nekhlyudov L., Goddard K.A.B., Davis R.L. (2012). Risk of heart failure in breast cancer patients after anthracycline and trastuzumab treatment: A retrospective cohort study. J. Natl. Cancer Inst..

[39] Ewer M.S., Ewer S.M. (2010). Troponin I provides insight into cardiotoxicity and the anthracycline-trastuzumab interaction. J. Clin. Oncol..

[40] de Korte M.A., de Vries E.G.E., Lub-de Hooge M.N., Jager P.L., Gietema J.A., van der Graaf W.T.A., Sluiter W.J., van Veldhuisen D.J., Suter T.M., Sleijfer D.T. (2007). 111Indium-trastuzumab visualises myocardial human epidermal growth factor receptor 2 expression shortly after anthracycline treatment but not during heart failure: A clue to uncover the mechanisms of trastuzumab-related cardiotoxicity. Eur. J. Cancer.

[41] Gaitanis G., Bassukas I.D. (2014). Intralesional bevacizumab as in-add adjuvant to immunocryosurgery for locally advanced basal cell carcinoma. J. Eur. Acad. Dermatol. Venereol..

[42] Schutz F.A.B., Je Y., Azzi G.R., Nguyen P.L., Choueiri T.K. (2011). Bevacizumab increases the risk of arterial ischemia: A large study in cancer patients with a focus on different subgroup outcomes. Ann. Oncol. Off. J. Eur. Soc. Med. Oncol..

[43] Ranpura V., Hapani S., Chuang J., Wu S. (2010). Risk of cardiac ischemia and arterial thromboembolic events with the angiogenesis inhibitor bevacizumab in cancer patients: A meta-analysis of randomized controlled trials. Acta Oncol..

[44] Choueiri T.K., Schutz F.A.B., Je Y., Rosenberg J.E., Bellmunt J. (2010). Risk of arterial thromboembolic events with sunitinib and sorafenib: A systematic review and meta-analysis of clinical trials. J. Clin. Oncol..

[45] Li Y., Gao Z.-H., Qu X.-J. (2015). The adverse effects of sorafenib in patients with advanced cancers. Basic Clin. Pharmacol. Toxicol..

[46] Chintalgattu V., Ai D., Langley R.R., Zhang J., Bankson J.A., Shih T.L., Reddy A.K., Coombes K.R., Daher I.N., Pati S. (2010). Cardiomyocyte PDGFR-beta signaling is an essential component of the mouse cardiac response to load-induced stress. J. Clin. Investig..

[47] Faderl S., Talpaz M., Estrov Z., O’Brien S., Kurzrock R., Kantarjian H.M. (1999). The biology of chronic myeloid leukemia. N. Engl. J. Med..

[48] O’Brien S.G., Guilhot F., Larson R.A., Gathmann I., Baccarani M., Cervantes F., Cornelissen J.J., Fischer T., Hochhaus A., Hughes T. (2003). Imatinib compared with interferon and low-dose cytarabine for newly diagnosed chronic-phase chronic myeloid leukemia. N. Engl. J. Med..

[49] Moslehi J.J., Deininger M. (2015). Tyrosine Kinase Inhibitor-Associated Cardiovascular Toxicity in Chronic Myeloid Leukemia. J. Clin. Oncol..

[50] Kerkelä R., Grazette L., Yacobi R., Iliescu C., Patten R., Beahm C., Walters B., Shevtsov S., Pesant S., Clubb F.J. (2006). Cardiotoxicity of the cancer therapeutic agent imatinib mesylate. Nat. Med..

[51] Varga Z.V., Ferdinandy P., Liaudet L., Pacher P. (2015). Drug-induced mitochondrial dysfunction and cardiotoxicity. Am. J. Physiol. Heart Circ. Physiol..

[52] Aghel N., Delgado D.H., Lipton J.H. (2017). Cardiovascular toxicities of BCR-ABL tyrosine kinase inhibitors in chronic myeloid leukemia: Preventive strategies and cardiovascular surveillance. Vasc. Health Risk Manag..

[53] Montani D., Seferian A., Savale L., Simonneau G., Humbert M. (2013). Drug-induced pulmonary arterial hypertension: A recent outbreak. Eur. Respir. Rev..

[54] Remsing Rix L.L., Rix U., Colinge J., Hantschel O., Bennett K.L., Stranzl T., Müller A., Baumgartner C., Valent P., Augustin M. (2009). Global target profile of the kinase inhibitor bosutinib in primary chronic myeloid leukemia cells. Leukemia.

[55] Anagnostou T., Litzow M.R. (2018). Spotlight on ponatinib in the treatment of chronic myeloid leukemia and Philadelphia chromosome-positive acute lymphoblastic leukemia: Patient selection and perspectives. Blood Lymphat. Cancer.

[56] Papaioannou N.E., Beniata O.V., Vitsos P., Tsitsilonis O., Samara P. (2016). Harnessing the immune system to improve cancer therapy. Ann. Transl. Med..

[57] Paulson K.G., Lahman M.C., Chapuis A.G., Brownell I. (2019). Immunotherapy for skin cancer. Int. Immunol..

[58] Mehta S.R., Suhag V., Semwal M., Sharma N. (2010). Radiotherapy: Basic Concepts and Recent Advances. Med. J. Armed Forces India.

[59] Locke J., Karimpour S., Young G., Lockett M.A., Perez C.A. (2001). Radiotherapy for epithelial skin cancer. Int. J. Radiat. Oncol. Biol. Phys..

[60] Amols H.I. (2008). New technologies in radiation therapy: Ensuring patient safety, radiation safety and regulatory issues in radiation oncology. Health Phys..

[61] Seibert J.A. (2004). X-ray imaging physics for nuclear medicine technologists. Part 1: Basic principles of X-ray production. J. Nucl. Med. Technol..

[62] Wang H., Wei J., Zheng Q., Meng L., Xin Y., Yin X., Jiang X. (2019). Radiation-induced heart disease: A review of classification, mechanism and prevention. Int. J. Biol. Sci..

[63] Cuomo J.R., Sharma G.K., Conger P.D., Weintraub N.L. (2016). Novel concepts in radiation-induced cardiovascular disease. World J. Cardiol..

[64] Schimmel K.J.M., Richel D.J., van den Brink R.B.A., Guchelaar H.-J. (2004). Cardiotoxicity of cytotoxic drugs. Cancer Treat. Rev..

[65] Rowinsky E.K., Eisenhauer E.A., Chaudhry V., Arbuck S.G., Donehower R.C. (1993). Clinical toxicities encountered with paclitaxel (Taxol). Semin. Oncol..

[66] Chong J.H., Ghosh A.K. (2019). Coronary Artery Vasospasm Induced by 5-fluorouracil: Proposed Mechanisms, Existing Management Options and Future Directions. Interv. Cardiol. (Lond. Engl.).

[67] Gavazzoni M., Vizzardi E., Gorga E., Bonadei I., Rossi L., Belotti A., Rossi G., Ribolla R., Metra M., Raddino R. (2018). Mechanism of cardiovascular toxicity by proteasome inhibitors: New paradigm derived from clinical and pre-clinical evidence. Eur. J. Pharmacol..

[68] Gianni L., Salvatorelli E., Minotti G. (2007). Anthracycline cardiotoxicity in breast cancer patients: Synergism with trastuzumab and taxanes. Cardiovasc. Toxicol..

[69] Zielinski C.C. (2003). Gemcitabine, anthracycline, and taxane combinations for advanced breast cancer. Oncology (Williston Park).

[70] Spur E.-M., Althof N., Respondek D., Klingel K., Heuser A., Overkleeft H.S., Voigt A. (2016). Inhibition of chymotryptic-like standard proteasome activity exacerbates doxorubicin-induced cytotoxicity in primary cardiomyocytes. Toxicology.

[71] Thavendiranathan P., Poulin F., Lim K.-D., Plana J.C., Woo A., Marwick T.H. (2014). Use of myocardial strain imaging by echocardiography for the early detection of cardiotoxicity in patients during and after cancer chemotherapy: A systematic review. J. Am. Coll. Cardiol..

[72] Armstrong G.T., Plana J.C., Zhang N., Srivastava D., Green D.M., Ness K.K., Daniel Donovan F., Metzger M.L., Arevalo A., Durand J.-B. (2012). Screening adult survivors of childhood cancer for cardiomyopathy: Comparison of echocardiography and cardiac magnetic resonance imaging. J. Clin. Oncol..

[73] Jones R.L., Miles D.W. (2005). Use of endomyocardial biopsy to assess anthracycline-induced cardiotoxicity. Lancet Oncol..

[74] Caspi O., Aronson D. (2019). Surviving Cancer without a Broken Heart. Rambam Maimonides Med. J..

[75] Frères P., Bouznad N., Servais L., Josse C., Wenric S., Poncin A., Thiry J., Moonen M., Oury C., Lancellotti P. (2018). Variations of circulating cardiac biomarkers during and after anthracycline-containing chemotherapy in breast cancer patients. BMC Cancer.

[76] Zhu J.N., Fu Y.H., Hu Z., Li W.Y., Tang C.M., Fei H.W., Yang H., Lin Q., Gou D.M., Wu S.L. (2017). Activation of miR-34a-5p/Sirt1/p66shc pathway contributes to doxorubicin-induced cardiotoxicity. Sci. Rep..

[77] Hu Y., Xia W., Hou M. (2018). Macrophage migration inhibitory factor serves a pivotal role in the regulation of radiation-induced cardiac senescencethrough rebalancing the microRNA-34a/sirtuin 1 signaling pathway. Int. J. Mol. Med..

[78] Lacombe J., Zenhausern F. (2017). Emergence of miR-34a in radiation therapy. Crit. Rev. Oncol. Hematol..

[79] Park B., Yee C., Lee K.-M. (2014). The effect of radiation on the immune response to cancers. Int. J. Mol. Sci..

[80] Grabie N., Gotsman I., DaCosta R., Pang H., Stavrakis G., Butte M.J., Keir M.E., Freeman G.J., Sharpe A.H., Lichtman A.H. (2007). Endothelial programmed death-1 ligand 1 (PD-L1) regulates CD8+ T-cell mediated injury in the heart. Circulation.

[81] Spallarossa P., Altieri P., Garibaldi S., Ghigliotti G., Barisione C., Manca V., Fabbi P., Ballestrero A., Brunelli C., Barsotti A. (2006). Matrix metalloproteinase-2 and -9 are induced differently by doxorubicin in H9c2 cells: The role of MAP kinases and NAD(P)H oxidase. Cardiovasc. Res..

[82] Eken S.M., Christersdottir T., Winski G., Sangsuwan T., Jin H., Chernogubova E., Pirault J., Sun C., Simon N., Winter H. (2019). miR-29b Mediates the Chronic Inflammatory Response in Radiotherapy-Induced Vascular Disease. JACC Basic Transl. Sci..

[83] Yin C., Salloum F.N., Kukreja R.C. (2009). A novel role of microRNA in late preconditioning: Upregulation of endothelial nitric oxide synthase and heat shock protein 70. Circ. Res..

[84] Dong S., Cheng Y., Yang J., Li J., Liu X., Wang X., Wang D., Krall T.J., Delphin E.S., Zhang C. (2009). MicroRNA expression signature and the role of microRNA-21 in the early phase of acute myocardial infarction. J. Biol. Chem..

[85] Roy S., Khanna S., Hussain S.-R.A., Biswas S., Azad A., Rink C., Gnyawali S., Shilo S., Nuovo G.J., Sen C.K. (2009). MicroRNA expression in response to murine myocardial infarction: miR-21 regulates fibroblast metalloprotease-2 via phosphatase and tensin homologue. Cardiovasc. Res..

[86] Thum T., Gross C., Fiedler J., Fischer T., Kissler S., Bussen M., Galuppo P., Just S., Rottbauer W., Frantz S. (2008). MicroRNA-21 contributes to myocardial disease by stimulating MAP kinase signalling in fibroblasts. Nature.

[87] Viczenczova C., Bacova B.S., Benova T.E., Kura B., Yin C., Weismann P., Kukreja R., Slezak J., Tribulova N. (2016). Myocardial connexin-43 and PKC signalling are involved in adaptation of the heart to irradiation-induced injury: Implication of miR-1 and miR-21. Gen. Physiol. Biophys..

[88] Kopcalic K., Petrovic N., Stanojkovic T.P., Stankovic V., Bukumiric Z., Roganovic J., Malisic E., Nikitovic M. (2019). Association between miR-21/146a/155 level changes and acute genitourinary radiotoxicity in prostate cancer patients: A pilot study. Pathol. Res. Pract..

[89] Tang Y., Zheng J., Sun Y., Wu Z., Liu Z., Huang G. (2009). MicroRNA-1 regulates cardiomyocyte apoptosis by targeting Bcl-2. Int. Heart J..

[90] Shan Z.X., Lin Q.X., Fu Y.H., Deng C.Y., Zhou Z.L., Zhu J.N., Liu X.Y., Zhang Y.Y., Li Y., Lin S.G. (2009). Upregulated expression of miR-1/miR-206 in a rat model of myocardial infarction. Biochem. Biophys. Res. Commun..

[91] Bostjancic E., Zidar N., Stajner D., Glavac D. (2010). MicroRNA miR-1 is up-regulated in remote myocardium in patients with myocardial infarction. Folia Biol..

[92] Tony H., Yu K., Qiutang Z. (2015). MicroRNA-208a Silencing Attenuates Doxorubicin Induced Myocyte Apoptosis and Cardiac Dysfunction. Oxid. Med. Cell. Longev..

[93] Oliveira-Carvalho V., Ferreira L.R.P., Bocchi E.A. (2015). Circulating mir-208a fails as a biomarker of doxorubicin-induced cardiotoxicity in breast cancer patients. J. Appl. Toxicol..

[94] Desai V.G., C Kwekel J., Vijay V., Moland C.L., Herman E.H., Lee T., Han T., Lewis S.M., Davis K.J., Muskhelishvili L. (2014). Early biomarkers of doxorubicin-induced heart injury in a mouse model. Toxicol. Appl. Pharmacol..

[95] Verjans R., Peters T., Beaumont F.J., van Leeuwen R., van Herwaarden T., Verhesen W., Munts C., Bijnen M., Henkens M., Diez J. (2018). MicroRNA-221/222 Family Counteracts Myocardial Fibrosis in Pressure Overload–Induced Heart Failure. Hypertension.

[96] Wang C., Wang S., Zhao P., Wang X., Wang J., Wang Y., Song L., Zou Y., Hui R. (2012). MiR-221 promotes cardiac hypertrophy in vitro through the modulation of p27 expression. J. Cell. Biochem..

[97] Nishimura H., Okazaki T., Tanaka Y., Nakatani K., Hara M., Matsumori A., Sasayama S., Mizoguchi A., Hiai H., Minato N. (2001). Autoimmune dilated cardiomyopathy in PD-1 receptor-deficient mice. Science.

[98] Watson C.J., Gupta S.K., O’Connell E., Thum S., Glezeva N., Fendrich J., Gallagher J., Ledwidge M., Grote-Levi L., McDonald K. (2015). MicroRNA signatures differentiate preserved from reduced ejection fraction heart failure. Eur. J. Heart Fail..

[99] Esplugas R., Arenas M., Serra N., Bellés M., Bonet M., Gascón M., Vallvé J.-C., Linares V. (2019). Effect of radiotherapy on the expression of cardiovascular disease-related miRNA-146a, -155, -221 and -222 in blood of women with breast cancer. PLoS ONE.

[100] Plein A., Fantin A., Ruhrberg C. (2014). Neuropilin regulation of angiogenesis, arteriogenesis, and vascular permeability. Microcirculation.

[101] Scott J.M., Khakoo A., Mackey J.R., Haykowsky M.J., Douglas P.S., Jones L.W. (2011). Modulation of anthracycline-induced cardiotoxicity by aerobic exercise in breast cancer: Current evidence and underlying mechanisms. Circulation.

[102] Brabletz S., Brabletz T. (2010). The ZEB/miR-200 feedback loop--a motor of cellular plasticity in development and cancer?. EMBO Rep..

[103] Beji S., Milano G., Scopece A., Cicchillitti L., Cencioni C., Picozza M., D’Alessandra Y., Pizzolato S., Bertolotti M., Spaltro G. (2017). Doxorubicin upregulates CXCR4 via miR-200c/ZEB1-dependent mechanism in human cardiac mesenchymal progenitor cells. Cell Death Dis..

[104] Simůnek T., Stérba M., Popelová O., Adamcová M., Hrdina R., Gersl V. (2009). Anthracycline-induced cardiotoxicity: Overview of studies examining the roles of oxidative stress and free cellular iron. Pharmacol. Rep..

[105] Damrot J., Nübel T., Epe B., Roos W.P., Kaina B., Fritz G. (2006). Lovastatin protects human endothelial cells from the genotoxic and cytotoxic effects of the anticancer drugs doxorubicin and etoposide. Br. J. Pharmacol..

[106] Magenta A., Cencioni C., Fasanaro P., Zaccagnini G., Greco S., Sarra-Ferraris G., Antonini A., Martelli F., Capogrossi M.C. (2011). miR-200c is upregulated by oxidative stress and induces endothelial cell apoptosis and senescence via ZEB1 inhibition. Cell Death Differ..

[107] Carlomosti F., D’Agostino M., Beji S., Torcinaro A., Rizzi R., Zaccagnini G., Maimone B., Di Stefano V., De Santa F., Cordisco S. (2017). Oxidative Stress-Induced miR-200c Disrupts the Regulatory Loop among SIRT1, FOXO1, and eNOS. Antioxid. Redox Signal..

[108] Potente M., Dimmeler S. (2008). Emerging roles of SIRT1 in vascular endothelial homeostasis. Cell Cycle.

[109] Hu X., Liu H., Wang Z., Hu Z., Li L. (2019). miR-200a Attenuated Doxorubicin-Induced Cardiotoxicity through Upregulation of Nrf2 in Mice. Oxid. Med. Cell. Longev..

[110] Vacchi-Suzzi C., Bauer Y., Berridge B.R., Bongiovanni S., Gerrish K., Hamadeh H.K., Letzkus M., Lyon J., Moggs J., Paules R.S. (2012). Perturbation of microRNAs in rat heart during chronic doxorubicin treatment. PLoS ONE.

[111] Zhang W.-C., Yang J.-H., Liu G.-H., Yang F., Gong J.-L., Jia M.-G., Zhang M.-J., Zhao L.-S. (2019). miR-34b/c regulates doxorubicin-induced myocardial cell injury through ITCH. Cell Cycle.

[112] Cvetković R.S., Scott L.J. (2005). Dexrazoxane: A review of its use for cardioprotection during anthracycline chemotherapy. Drugs.

[113] Zhou S., Chen H.Z., Wan Y.Z., Zhang Q.J., Wei Y.S., Huang S., Liu J.J., Lu Y.B., Zhang Z.Q., Yang R.F. (2011). Repression of P66Shc expression by SIRT1 contributes to the prevention of hyperglycemia-induced endothelial dysfunction. Circ. Res..

[114] Bonfini L., Migliaccio E., Pelicci G., Lanfrancone L., Pelicci P.G. (1996). Not all Shc’s roads lead to Ras. Trends. Biochem. Sci..

[115] Tarrio M.L., Grabie N., Bu D., Sharpe A.H., Lichtman A.H. (2012). PD-1 protects against inflammation and myocyte damage in T cell-mediated myocarditis. J. Immunol..

[116] Baban B., Liu J.Y., Qin X., Weintraub N.L., Mozaffari M.S. (2015). Upregulation of Programmed Death-1 and Its Ligand in Cardiac Injury Models: Interaction with GADD153. PLoS ONE.

[117] Beg M.S., Brenner A.J., Sachdev J., Borad M., Kang Y.-K., Stoudemire J., Smith S., Bader A.G., Kim S., Hong D.S. (2017). Phase I study of MRX34, a liposomal miR-34a mimic, administered twice weekly in patients with advanced solid tumors. Investig. New Drugs.

[118] Jing X., Yang J., Jiang L., Chen J., Wang H. (2018). MicroRNA-29b Regulates the Mitochondria-Dependent Apoptotic Pathway by Targeting Bax in Doxorubicin Cardiotoxicity. Cell. Physiol. Biochem..

[119] Leger K.J., Leonard D., Nielson D., de Lemos J.A., Mammen P.P.A., Winick N.J. (2017). Circulating microRNAs: Potential Markers of Cardiotoxicity in Children and Young Adults Treated With Anthracycline Chemotherapy. J. Am. Heart Assoc..

[120] van Rooij E., Sutherland L.B., Thatcher J.E., DiMaio J.M., Naseem R.H., Marshall W.S., Hill J.A., Olson E.N. (2008). Dysregulation of microRNAs after myocardial infarction reveals a role of miR-29 in cardiac fibrosis. Proc. Natl. Acad. Sci. USA.

[121] Zile M.R., Mehurg S.M., Arroyo J.E., Stroud R.E., DeSantis S.M., Spinale F.G. (2011). Relationship between the temporal profile of plasma microRNA and left ventricular remodeling in patients after myocardial infarction. Circ. Cardiovasc. Genet..

[122] Roncarati R., Viviani Anselmi C., Losi M.A., Papa L., Cavarretta E., Da Costa Martins P., Contaldi C., Saccani Jotti G., Franzone A., Galastri L. (2014). Circulating miR-29a, among other up-regulated microRNAs, is the only biomarker for both hypertrophy and fibrosis in patients with hypertrophic cardiomyopathy. J. Am. Coll. Cardiol..

[123] Liu Y., Taylor N.E., Lu L., Usa K., Cowley A.W., Ferreri N.R., Yeo N.C., Liang M. (2010). Renal medullary microRNAs in Dahl salt-sensitive rats: miR-29b regulates several collagens and related genes. Hypertension (Dallas Tex. 1979).

[124] Kizaki K., Ito R., Okada M., Yoshioka K., Uchide T., Temma K., Mutoh K., Uechi M., Hara Y. (2006). Enhanced gene expression of myocardial matrix metalloproteinases 2 and 9 after acute treatment with doxorubicin in mice. Pharmacol. Res..

[125] Dinh T.-K.T., Fendler W., Chałubińska-Fendler J., Acharya S.S., O’Leary C., Deraska P.V., D’Andrea A.D., Chowdhury D., Kozono D. (2016). Circulating miR-29a and miR-150 correlate with delivered dose during thoracic radiation therapy for non-small cell lung cancer. Radiat. Oncol..

[126] Roca-Alonso L., Castellano L., Mills A., Dabrowska A.F., Sikkel M.B., Pellegrino L., Jacob J., Frampton A.E., Krell J., Coombes R.C. (2015). Myocardial MiR-30 downregulation triggered by doxorubicin drives alterations in β-adrenergic signaling and enhances apoptosis. Cell Death Dis..

[127] Lai L., Chen J., Wang N., Zhu G., Duan X., Ling F. (2017). MiRNA-30e mediated cardioprotection of ACE2 in rats with Doxorubicin-induced heart failure through inhibiting cardiomyocytes autophagy. Life Sci..

[128] Oltvai Z.N., Milliman C.L., Korsmeyer S.J. (1993). Bcl-2 heterodimerizes in vivo with a conserved homolog, Bax, that accelerates programmed cell death. Cell.

[129] Zhou F., Lu X., Zhang X. (2018). Serum miR-30c Level Predicted Cardiotoxicity in Non-small Cell Lung Cancer Patients Treated with Bevacizumab. Cardiovasc. Toxicol..

[130] Tong Z., Jiang B., Wu Y., Liu Y., Li Y., Gao M., Jiang Y., Lv Q., Xiao X. (2015). MiR-21 Protected Cardiomyocytes against Doxorubicin-Induced Apoptosis by Targeting BTG2. Int. J. Mol. Sci..

[131] Simone N.L., Soule B.P., Ly D., Saleh A.D., Savage J.E., Degraff W., Cook J., Harris C.C., Gius D., Mitchell J.B. (2009). Ionizing radiation-induced oxidative stress alters miRNA expression. PLoS ONE.

[132] Xi J., Huang Q., Wang L., Ma X., Deng Q., Kumar M., Zhou Z., Li L., Zeng Z., Young K.H. (2018). miR-21 depletion in macrophages promotes tumoricidal polarization and enhances PD-1 immunotherapy. Oncogene.

[133] Dorn G.W. (2010). MicroRNAs: Redefining Mechanisms in Cardiac Disease. J. Cardiovasc. Pharmacol..

[134] Chen J.F., Mandel E.M., Thomson J.M., Wu Q., Callis T.E., Hammond S.M., Conlon F.L., Wang D.Z. (2006). The role of microRNA-1 and microRNA-133 in skeletal muscle proliferation and differentiation. Nat. Genet..

[135] Zhao Y., Samal E., Srivastava D. (2005). Serum response factor regulates a muscle-specific microRNA that targets Hand2 during cardiogenesis. Nature.

[136] Cheng Y., Tan N., Yang J., Liu X., Cao X., He P., Dong X., Qin S., Zhang C. (2010). A translational study of circulating cell-free microRNA-1 in acute myocardial infarction. Clin. Sci. (Lond.).

[137] Nishimura Y., Kondo C., Morikawa Y., Tonomura Y., Torii M., Yamate J., Uehara T. (2015). Plasma miR-208 as a useful biomarker for drug-induced cardiotoxicity in rats. J. Appl. Toxicol..

[138] Rigaud V.O.-C., Ferreira L.R.P., Ayub-Ferreira S.M., Ávila M.S., Brandão S.M.G., Cruz F.D., Santos M.H.H., Cruz C.B.B.V., Alves M.S.L., Issa V.S. (2017). Circulating miR-1 as a potential biomarker of doxorubicin-induced cardiotoxicity in breast cancer patients. Oncotarget.

[139] Kura B., Yin C., Frimmel K., Krizak J., Okruhlicova L., Kukreja R.C., Slezak J. (2016). Changes of microRNA-1, -15b and -21 levels in irradiated rat hearts after treatment with potentially radioprotective drugs. Physiol. Res..

[140] Lu Y., Zhang Y., Shan H., Pan Z., Li X., Li B., Xu C., Zhang B., Zhang F., Dong D. (2009). MicroRNA-1 downregulation by propranolol in a rat model of myocardial infarction: A new mechanism for ischaemic cardioprotection. Cardiovasc. Res..

[141] McCarthy J.J., Esser K.A. (2007). MicroRNA-1 and microRNA-133a expression are decreased during skeletal muscle hypertrophy. J. Appl. Physiol..

[142] Xu C., Lu Y., Pan Z., Chu W., Luo X., Lin H., Xiao J., Shan H., Wang Z., Yang B. (2007). The muscle-specific microRNAs miR-1 and miR-133 produce opposing effects on apoptosis by targeting HSP60, HSP70 and caspase-9 in cardiomyocytes. J. Cell Sci..

[143] Ji X., Takahashi R., Hiura Y., Hirokawa G., Fukushima Y., Iwai N. (2009). Plasma miR-208 as a Biomarker of Myocardial Injury. Clin. Chem..

[144] Wan Q., Xu T., Ding W., Zhang X., Ji X., Yu T., Yu W., Lin Z., Wang J. (2018). miR-499-5p Attenuates Mitochondrial Fission and Cell Apoptosis via p21 in Doxorubicin Cardiotoxicity. Front. Genet..

[145] Garofalo M., Quintavalle C., Romano G., Croce C.M., Condorelli G. (2012). miR221/222 in cancer: Their role in tumor progression and response to therapy. Curr. Mol. Med..

[146] Liu X., Cheng Y., Yang J., Xu L., Zhang C. (2012). Cell-specific effects of miR-221/222 in vessels: Molecular mechanism and therapeutic application. J. Mol. Cell. Cardiol..

[147] Su M., Wang J., Wang C., Wang X., Dong W., Qiu W., Wang Y., Zhao X., Zou Y., Song L. (2015). MicroRNA-221 inhibits autophagy and promotes heart failure by modulating the p27/CDK2/mTOR axis. Cell Death Differ..

[148] Lieb V., Weigelt K., Scheinost L., Fischer K., Greither T., Marcou M., Theil G., Klocker H., Holzhausen H.-J., Lai X. (2018). Serum levels of miR-320 family members are associated with clinical parameters and diagnosis in prostate cancer patients. Oncotarget.

[149] Tao L., Bei Y., Zhou Y., Xiao J., Li X. (2015). Non-coding RNAs in cardiac regeneration. Oncotarget.

[150] Feng B., Chakrabarti S. (2012). miR-320 Regulates Glucose-Induced Gene Expression in Diabetes. ISRN Endocrinol..

[151] Yin Z., Zhao Y., Li H., Yan M., Zhou L., Chen C., Wang D.W. (2016). miR-320a mediates doxorubicin-induced cardiotoxicity by targeting VEGF signal pathway. Aging (Albany. N.Y.).

[152] Ambler G.R., Johnston B.M., Maxwell L., Gavin J.B., Gluckman P.D. (1993). Improvement of doxorubicin induced cardiomyopathy in rats treated with insulin-like growth factor I. Cardiovasc. Res..

[153] Wang L., Ma W., Markovich R., Chen J.W., Wang P.H. (1998). Regulation of cardiomyocyte apoptotic signaling by insulin-like growth factor I. Circ. Res..

[154] Menna P., Salvatorelli E. (2017). Primary Prevention Strategies for Anthracycline Cardiotoxicity: A Brief Overview. Chemotherapy.

[155] Fraguas-Sánchez A.I., Martín-Sabroso C., Fernández-Carballido A., Torres-Suárez A.I. (2019). Current status of nanomedicine in the chemotherapy of breast cancer. Cancer Chemother. Pharmacol..

[156] Singh C.K., Siddiqui I.A., El-Abd S., Mukhtar H., Ahmad N. (2016). Combination chemoprevention with grape antioxidants. Mol. Nutr. Food Res..

[157] Kirkham A.A., Davis M.K. (2015). Exercise Prevention of Cardiovascular Disease in Breast Cancer Survivors. J. Oncol..

[158] Herman E.H., Ferrans V.J., Myers C.E., Van Vleet J.F. (1985). Comparison of the effectiveness of (+/-)-1,2-bis(3,5-dioxopiperazinyl-1-yl)propane (ICRF-187) and N-acetylcysteine in preventing chronic doxorubicin cardiotoxicity in beagles. Cancer Res..

[159] Lenčová-Popelová O., Jirkovský E., Jansová H., Jirkovská-Vávrová A., Vostatková-Tichotová L., Mazurová Y., Adamcová M., Chládek J., Hroch M., Pokorná Z. (2016). Cardioprotective effects of inorganic nitrate/nitrite in chronic anthracycline cardiotoxicity: Comparison with dexrazoxane. J. Mol. Cell. Cardiol..

[160] Matsui H., Morishima I., Numaguchi Y., Toki Y., Okumura K., Hayakawa T. (1999). Protective effects of carvedilol against doxorubicin-induced cardiomyopathy in rats. Life Sci..

[161] de Nigris F., Rienzo M., Schiano C., Fiorito C., Casamassimi A., Napoli C. (2008). Prominent cardioprotective effects of third generation beta blocker nebivolol against anthracycline-induced cardiotoxicity using the model of isolated perfused rat heart. Eur. J. Cancer.

[162] Eindhoven J.A., van den Bosch A.E., Jansen P.R., Boersma E., Roos-Hesselink J.W. (2012). The usefulness of brain natriuretic peptide in complex congenital heart disease: A systematic review. J. Am. Coll. Cardiol..

[163] Guglin M., Krischer J., Tamura R., Fink A., Bello-Matricaria L., McCaskill-Stevens W., Munster P.N. (2019). Randomized Trial of Lisinopril Versus Carvedilol to Prevent Trastuzumab Cardiotoxicity in Patients With Breast Cancer. J. Am. Coll. Cardiol..

[164] Gao R., Zhang J., Cheng L., Wu X., Dong W., Yang X., Li T., Liu X., Xu Y., Li X. (2010). A Phase II, randomized, double-blind, multicenter, based on standard therapy, placebo-controlled study of the efficacy and safety of recombinant human neuregulin-1 in patients with chronic heart failure. J. Am. Coll. Cardiol..

[165] Jabbour A., Hayward C.S., Keogh A.M., Kotlyar E., McCrohon J.A., England J.F., Amor R., Liu X., Li X.Y., Zhou M.D. (2011). Parenteral administration of recombinant human neuregulin-1 to patients with stable chronic heart failure produces favourable acute and chronic haemodynamic responses. Eur. J. Heart Fail..

[166] Jay S.M., Murthy A.C., Hawkins J.F., Wortzel J.R., Steinhauser M.L., Alvarez L.M., Gannon J., Macrae C.A., Griffith L.G., Lee R.T. (2013). An engineered bivalent neuregulin protects against doxorubicin-induced cardiotoxicity with reduced proneoplastic potential. Circulation.

[167] Milano G., Biemmi V., Lazzarini E., Balbi C., Ciullo A., Bolis S., Ameri P., Di Silvestre D., Mauri P., Barile L. (2019). Intravenous administration of cardiac progenitor cell-derived exosomes protects against doxorubicin/trastuzumab-induced cardiac toxicity. Cardiovasc. Res..

